# Fabrication and Properties of Functional Coatings Under Extreme Conditions

**DOI:** 10.3390/ma18225231

**Published:** 2025-11-19

**Authors:** Chaoyang Chen, Lingjie Chen, Guoqin Cao

**Affiliations:** 1 State Centre for International Cooperation on Designer Low-Carbon & Environmental Materials, School of Materials Science and Engineering, Zhengzhou University, Zhengzhou 450001, China; 2 National Key Laboratory of Marine Corrosion and Protection, Luoyang Ship Material Research Institute, Luoyang 471023, China

The advancement of aerospace, marine engineering, and advanced energy systems hinges on material performance under extreme service conditions (intense thermal exposure, severe corrosion, high mechanical stress) [1,2]. Conventional materials/coatings fall short here—limitations in thermal stability, corrosion resistance, and mechanical integrity under multi-factor stress create performance gaps and operational risks, even leading to catastrophic equipment damage and economic loss. Thus, developing next-generation functional coatings (with enhanced durability, multifunctionality, adaptive protection) is both a scientific priority and a technological imperative for these sectors [3,4,5]. Future progress will depend on innovative material architectures and advanced fabrication technologies: key directions include self-healing coatings (based on stimuli-responsive micro/nanocontainers), multifunctional materials (integrating corrosion inhibition, lubricity), and emerging synthesis methods supported by advanced theoretical modeling. These developments are critical for enabling applications at the limits of material capability [6,7,8].

This Special Issue focuses particularly on service behavior under extreme nuclear, aerospace, and marine environments, alongside the preparation and implementation of emerging materials—including nanocomposites, multilayer architectures, high-entropy alloys, and high-entropy ceramics. These advanced materials have been extensively investigated in recent years due to their exceptional structural stability and environmental adaptability [9,10,11]. Contributions addressing the characterization of complex oxidation processes, interfacial interactions, and the correlation between composition, structure, and in-service stability are especially welcome.

The ten articles included in this Special Issue collectively establish an integrated research chain spanning “fabrication–characterization–application under extreme conditions”. Each contribution addresses specific industrial challenges while deepening fundamental insights. In the context of extreme wear environments, such as those encountered by mining machinery, molds, and cutting tools subjected to high loads and abrasive dust, two studies are particularly noteworthy. Piotrowska et al. [contribution 1] examined the tribological performance of diamond-like carbon (DLC) coatings of the a-C:H type, deposited by plasma-assisted chemical vapor deposition, in comparison with conventional 100Cr6 steel in mine conveyor belt bearings. Their results indicated that the DLC coating reduced the friction coefficient by more than 30%, decreased the wear rate to one-fifth that of 100Cr6 steel, and doubled the mean time between failures, thereby substantially lowering equipment downtime losses. In another study, Hsu et al. [contribution 2] fabricated (Ti, Cr, Cu, Al, Si)N multi-element nitride coatings on AISI H13 tool steel via cathodic arc deposition. By optimizing the N_2_/Ar flow ratio to 2, they obtained a dense solid-solution structure exhibiting a hardness of 28 GPa, i.e., three times that of the substrate, along with interfacial adhesion exceeding 50 N and a 40% reduction in wear loss during high-temperature cutting operations. Notably, these performance gains were achieved while maintaining low surface roughness (Ra < 0.5 μm), essential for preserving machining precision.

For applications in high-temperature and nuclear environments, such as nuclear accident-tolerant fuel claddings and aero-engine blades, where exceptional oxidation resistance is critical, a collaborative study conducted by Dr. Guoqin Cao [contribution 3] investigated Al-doped TiCrZrNbTa refractory high-entropy alloy (RHEA) coatings. These coatings were deposited onto Zry-4 substrates via magnetron sputtering. Oxidation tests conducted in high-temperature steam at 1000–1100 °C revealed that a composition of 10 at.% Al promoted the formation of a dense composite oxide scale (Al_2_O_3_–TiO_2_–Cr_2_O_3_), which reduced the oxidation weight gain to just one-tenth of that observed in Al-free coatings, while maintaining interfacial adhesion strength above 45 MPa without evidence of cracking. In contrast, Al concentrations equal to or exceeding 17 at.% resulted in the formation of brittle Al-rich phases, which accelerated both oxidation and spallation. These findings delineate a critical “Al threshold” in the compositional design of high-entropy alloy coatings for extreme service conditions.

Addressing corrosion challenges in marine and acidic environments, where high-salt sprays and industrial acids degrade metallic substrates, three studies in this issue present innovative coating solutions. Wenqiang Li et al. [contribution 4] fabricated Fe_60_Co_10−x_Ni_15_Cr_15_Si_x_ coatings on Q235 steel via a combined aerosol powder synthesis and laser cladding route, demonstrating that 4 at.% Si optimally balanced corrosion resistance and cost—achieving a corrosion current density only 1/20 that of bare Q235 steel in 3.5% NaCl solution, with a 15% cost reduction compared to Si-free counterparts. In contrast, higher Si content (8 at.%) compromised performance due to its intrinsic poor corrosion resistance. Aleksandra Kucharczyk-Kotlewska et al. [contribution 5] developed VTMS/PEDOT/PMo12 composite coatings for X20Cr13 and 41Cr4 steels, which reduced the corrosion rate by over 90% in pH 2 acidic medium—attributed to PMo_12_’s ion barrier functionality and PEDOT’s ability to facilitate passive film formation. Meanwhile, Agata Dudek et al. [contribution 6] engineered VTMS/HAp/VTMS/VTMS multilayer coatings on Grade 2 titanium using a sol–gel process, achieving interfacial adhesion of 30 N, a corrosion current density 50 times lower than that of pure titanium in simulated body fluid, and enhanced cytocompatibility via hydroxyapatite (HAp) incorporation—offering an integrated coating strategy for biomedical implants such as artificial joints.

In a study that bridges corrosion protection and novel fabrication methods, Pelucchi et al. [contribution 7] investigated a direct electrochemical route for depositing graphene onto steel substrates via cyclic voltammetry. This innovative approach bypasses the traditional, environmentally taxing graphene oxide (GO) synthesis step, enabling in situ oxidation and reduction in graphene flakes directly on the metal surface. While the coating quality of this direct method currently does not surpass that of conventional GO-based coatings, it presents a significant advancement in process sustainability and simplification, pointing toward greener fabrication routes for protective graphene coatings.

Beyond traditional protection, two articles expand functional coatings to optics and textiles. Daofeng Zhu et al. [contribution 8] synthesized amphiphilic polyacrylate (PMLEA) via solution radical copolymerization, optimizing the LA/ER-10 mass ratio to 1:2 to create a peelable coating that completely removes dust, grease, and fingerprints from quartz glass without residue, while maintaining excellent mechanical properties (2.57 MPa tensile strength, 183% elongation at break) and temperature stability (−20–80 °C) for high-precision optical lenses and spacecraft sensors. Mariia Svyntkivska et al. [contribution 9] coated cotton and PET fabrics with graphene oxide (GO) via padding, using quercetin (a natural flavonoid) for thermal reduction—achieving surface resistances as low as 240 kΩ/sq (PET) and 750 kΩ/sq (cotton), hydrophobicity (163° and 147° water contact angles, respectively), and over 99% antibacterial rates, supporting smart and medical textiles.

Finally, addressing ceramic-metal bonding challenges, which are critical for solid oxide fuel cells and high-temperature sensors, Shu-Wei Chang et al. [contribution 10] deposited 1.5–6 μm Cu-Ti coatings (70.2Cu-29.8Ti at.%) on ZrO_2_ and Crofer via vacuum sputtering then used silver-based brazing for reactive air brazing (RAB). A 3 μm coating formed “Ti-O-Zr” chemical bonds with ZrO_2_ and solid solutions with Crofer, eliminating interfacial defects and maintaining hermeticity (no leakage) for 28 h at 2 psig (room temperature) and 24 h at 600 °C—exceeding industry standards and advancing fuel cell industrialization.

In conclusion, this Special Issue compiles the latest advances in functional coatings for extreme environments, integrating material innovation, process optimization, and mechanistic studies to address industrial challenges and outline future research directions—such as coatings for multi-factor coupled extreme conditions, atomic-level interface regulation, and coating-substrate synergy. Academically, it establishes quantitative thresholds for multi-component coatings, develops eco-friendly processes, and builds a robust performance prediction framework. Industrially, it meets critical needs across five strategic sectors: energy, manufacturing, marine, medical, and aerospace. As an open-access resource, it promotes global collaboration and technology transfer. Dr. Guoqin Cao, the Guest Editor, wishes to express gratitude to all authors, reviewers, and the *Materials* editorial team. “We aim for this Special Issue to serve as a key academic platform for the extreme-environment coating community, driving interdisciplinary collaboration and innovation to advance materials protection for global high-end equipment.” The Second Edition of Fabrication and Properties of Functional Coatings Under Extreme Conditions is on https://www.mdpi.com/journal/materials/special_issues/HV8A3HG7YG (accessed on 16 November 2025).
